# Subject-Specific Automatic Reconstruction of White Matter Tracts

**DOI:** 10.1007/s10278-023-00883-0

**Published:** 2023-08-03

**Authors:** Stephan Meesters, Maud Landers, Geert-Jan Rutten, Luc Florack

**Affiliations:** 1 Department of Mathematics & Computer Science, Eindhoven University of Technology, Eindhoven, The Netherlands; 2 Department of Neurosurgery, Elisabeth-Tweesteden Hospital, Tilburg, The Netherlands

**Keywords:** MRI, Tractography, Brain tumor, Reliability, Automated pipeline

## Abstract

MRI-based tractography is still underexploited and unsuited for routine use in brain tumor surgery due to heterogeneity of methods and functional–anatomical definitions and above all, the lack of a turn-key system. Standardization of methods is therefore desirable, whereby an objective and reliable approach is a prerequisite before the results of any automated procedure can subsequently be validated and used in neurosurgical practice. In this work, we evaluated these preliminary but necessary steps in healthy volunteers. Specifically, we evaluated the robustness and reliability (i.e., test–retest reproducibility) of tractography results of six clinically relevant white matter tracts by using healthy volunteer data (*N* = 136) from the Human Connectome Project consortium. A deep learning convolutional network-based approach was used for individualized segmentation of regions of interest, combined with an evidence-based tractography protocol and appropriate post-tractography filtering. Robustness was evaluated by estimating the consistency of tractography probability maps, i.e., averaged tractograms in normalized space, through the use of a hold-out cross-validation approach. No major outliers were found, indicating a high robustness of the tractography results. Reliability was evaluated at the individual level. First by examining the overlap of tractograms that resulted from repeatedly processed identical MRI scans (*N* = 10, 10 iterations) to establish an upper limit of reliability of the pipeline. Second, by examining the overlap for subjects that were scanned twice at different time points (*N* = 40). Both analyses indicated high reliability, with the second analysis showing a reliability near the upper limit. The robust and reliable subject-specific generation of white matter tracts in healthy subjects holds promise for future validation of our pipeline in a clinical population and subsequent implementation in brain tumor surgery.

## Introduction

Knowledge of patient-specific white matter tracts is imperative for brain surgical procedures to minimize the loss of sensorimotor, language, or cognitive functions. Diffusion-weighted tractography methods have enabled in vivo reconstruction of white matter structures and already yield valuable clinical information regarding the relationship of peritumoral tracts to glioma [1]. However, tractography is still underexploited and not very well suited for use in routine clinical practice. Data analysis is complex and requires dedicated software as well as a skilled and informed user. Besides a variety of conceptual possibilities and technical parameters to choose from, there is also no definite clinical consensus on the functional–anatomical definitions of the various white matter tracts [2, 3]. Schilling et al. recently performed a study whereby 42 independent teams were given similar sets of whole-brain streamlines [4]. The authors observed a very large variability in the segmentation of tracts between teams and concluded that this is to a large extent caused by user-variability and the lack of a consistent framework for defining tracts. This stresses the need for standardized methods and the use of automated pipelines in clinical practice.

Semi-automatic procedures have initially been developed to reduce this variability [5, 6]. Over the past decade, several studies have gone one step further and focused on automating the entire process, including data acquisition, detection, and correction of abnormalities in the diffusion data, reconstruction of white matter tracts, and visualization of results [7–12]. Most of these studies generate streamlines via anatomically defined regions of interest (ROIs). A common approach is to use a brain atlas that is defined in a normalized space (such as the MNI-152 template), whereby the standardized brain has predefined (sub)cortical brain regions [13]. While this approach has the advantage of speed, involving usually a linear or non-linear transformation from atlas to patient space, its ability to account for anatomical inter-subject variability is limited [14]. This shortfall becomes especially prominent in patients with brain tumors, such as low- or high-grade gliomas, where local deformation and/or infiltration of the brain due to tumor growth can lead to significant errors in the co-registration process or to tissue misclassification [15–17].

With the introduction of processing toolboxes for neuroimaging, more accurate subject-specific segmentations were made. Examples are FreeSurfer and MAPER, which use extensive image techniques from the computer vision field such as the Bayesian approach with anatomical priors, or the Markov Random Field approach [18, 19]. However, these approaches are computationally very demanding, and its runtime would likely become a limiting factor for a processing pipeline deployed for clinical use, where a timely processing is important. In recent years, there has been a rise of neural network-based segmentations of brain structures using deep convolutional neural networks (DCNN). These DCNNs have shown to outperform classical registration-based approaches in both segmentation accuracy and computation time (excluding any one-time network training steps) [20]. Examples of DCNN-based frameworks capable of segmenting over 100 cortical and subcortical structures are BrainSegNet [21], DeepNAT [22], SLANT [23], QuickNAT [24], PSACNN [25], Assemblynet [26], FastSurfer [27], and ACEnet [28].

As with any technique, baseline performance and characteristics should preferably be tested in healthy subjects and be of sufficient quality before, in our case, pipeline results can be used in patients with brain tumors and subsequent validated against clinical expert opinion [29]. In this study, we focus on this first step and investigate whether an automated pipeline (i.e., without any user intervention) can provide robust and reliable tractography results in healthy subjects. Note that in this study, we are not validating pipeline results in patients (for example, against intraoperative subcortical electrical stimulation mapping). We define robustness as the ability of the pipeline to produce tracts in every subject (i.e., there should be no false-negatives in healthy subjects) and to do so with a minimal number of obvious outliers as judged against generally accepted anatomical knowledge (i.e., we aim for a low number of false-positives). We define reliability as test–retest reproducibility of pipeline results at the level of a single subject.

Four different experiments are performed with data of 136 healthy subjects that were randomly selected from the Human Connectome Project. We have opted for the SLANT (Spatially Localized Atlas Network Tiles) algorithm, which has an excellent segmentation performance and allows for implementation inside a Docker container for fast pipeline integration [23]. Over the past years, we have regularly processed brain tumor patients with our pipeline and compared results against clinical techniques and expertise, among others intraoperative electrical stimulation mapping and results from commercially available software (Medtronic Stealth Viz) [30]. After a period of feedback and optimization in approximately fifty cases, we became convinced that our automatically generated pipeline results had significant clinical relevance and decided (in retrospect) to describe its characteristics in terms of performance in a healthy population. As such, in anticipation of further future validation and use of the pipeline in brain tumor patients, six clinically relevant white matter tracts were tested in the current study [1, 31, 32]. In the first experiment, tractography results of all healthy subjects are combined into tractogram probability maps to capture the variability that results from various subject-specific and pipeline-specific sources along the pipeline, such as anatomical differences and the variability that is inherent to probabilistic tractography. In the second experiment, a hold-out cross validation is performed to quantify the consistency of individual results. Such a procedure can signal whether substantial deviations from the mean are present, whereby we assume that in a population of healthy subjects, these outliers represent false-positives. To assess reliability, two additional experiments were performed. In a third experiment, diffusion-weighted scans from 10 randomly picked healthy subjects were processed 10 times to quantify the influence of non-deterministic elements in the pipeline (namely the probabilistic tractography algorithm) and to establish an overall upper limit of reliability. To assess the effect of physiological and non-physiological sources of noise on tractograms, we analyzed data from 40 subjects that were scanned twice at different occasions (experiment 4).

## Material and Methods

**Fig. 1:**
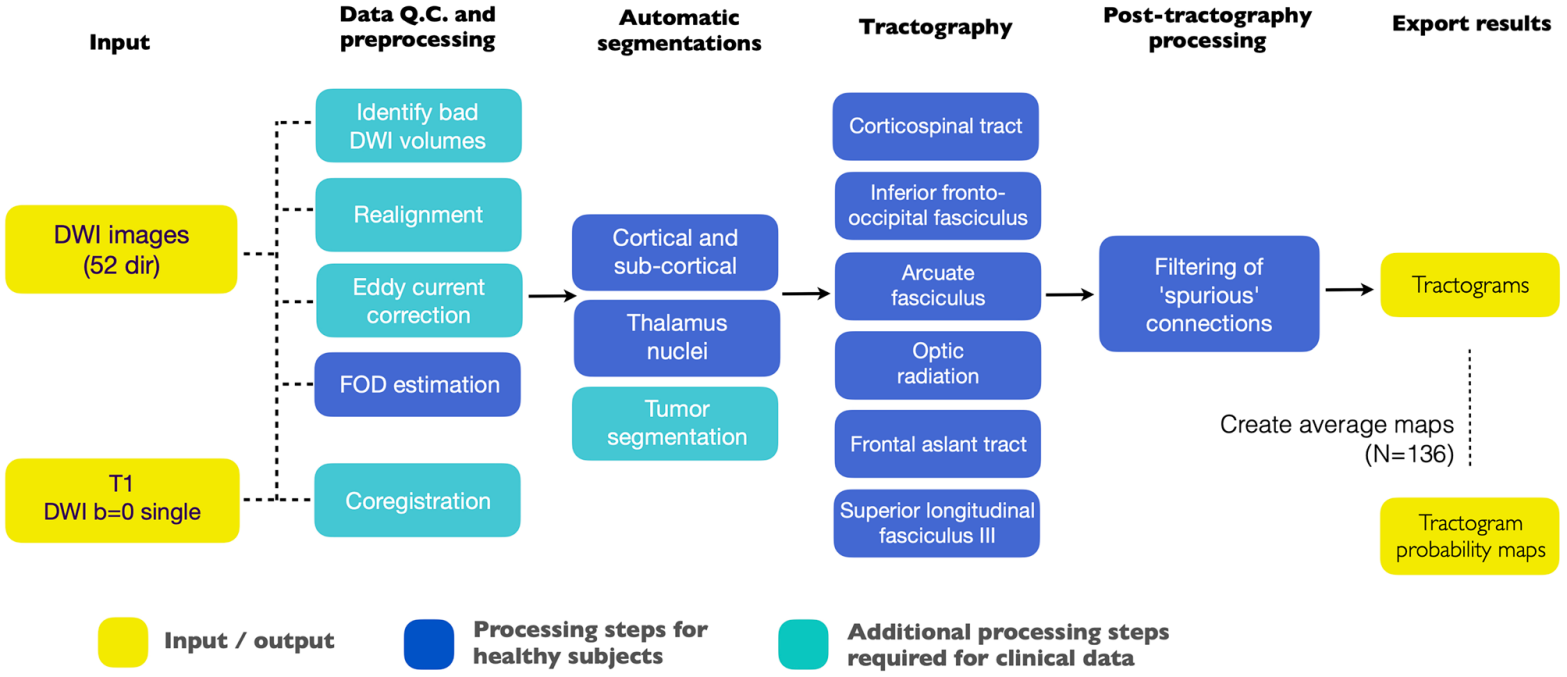
Overview of the automatic pipeline, showing the steps from processing the initial input data (the “Imaging Data and Preprocessing” section), automatic segmentation of regions of interest (the “Automatic segmentations of regions of interest” section), tractography of six white matter tracts (the “Tractography Algorithm and Parameters” and “Anatomical Definitions of ROIs” sections), tractography filtering (the “Post-tractography Filtering” section) and creation of respectively tractograms and tractogram probability maps (the “Experiments” section). Steps that were included in the pipeline for healthy subjects are color-coded in blue, and additional steps required for patient data are shown in green. The input image data and the output tractograms and associated maps are colored in yellow

The automated pipeline is schematically outlined in Fig. 1. Details on tractography of the six white matter tracts are given in the “Anatomical Definitions of ROIs” section. A distinction is made using color-coding regarding the processing steps used for healthy volunteer data, which will be the data considered in this study, and the additional processing steps that are required for clinical data (to be formally evaluated in future studies).

### Imaging Data and Preprocessing

Magnetic resonance imaging (MRI) data from the Human Connectome Project (HCP) were used [33]. The WU-Minn HCP Data S1200 release contains structural and functional MRI data that was acquired on a 3 T Connectom Skyra (Siemens, Erlangen) MRI scanner equipped with customized hardware, namely a gradient coil and gradient power amplifiers that allow for a maximum gradient strength of 100 mT/m that especially benefits the quality of the diffusion MRI (dMRI) scans. The current study made use of the T1 anatomical scan and the dMRI scans.

The dMRI scans were acquired with an isotropic spatial resolution of 1.25 mm, three diffusion weighting values (*b* = 1000, 2000, 3000 s/mm^2^) and 90 diffusion directions. The structural T1 scan was acquired at 0.7 mm isotropic. The HCP provides for each subject a preprocessed T1 anatomical scan that was corrected for readout distortions and a preprocessed DWI image, for which motion correction, susceptibility distortion and eddy-current correction have been applied [34].

For the current study, we selected 136 unrelated subjects from the S1200 release and used the preprocessed scans [33]. Steps were taken to make the high-quality HCP dataset more akin to a dataset acquired from a clinical protocol, which typically involves a shorter acquisition time and consequently has a lower number of diffusion directions, only a single shell, and/or a lower resolution. To this end, the T1 anatomical scan was resampled to 1 mm isotropic, and the dMRI scans were resampled to 2 mm isotropic and reduced to a single shell with a diffusion weighting of *b* = 2000. In addition to the scans used from the S1200 release, 40 of the included subjects were scanned on a separate occasion (range 1–11 months; average 4.7 ± 2 months) with the exact same scanning protocol, hardware, and preprocessing scripts as part of the S1200 test–retest interval update dataset. The dMRI was processed using MRTrix3 to create a fiber orientation density function (fODF), which is a modelling technique to represent the arrangement of white matter fibers and has the ability to resolve regions of crossing fiber configurations [35, 36]. To this end, the dwi2response routine was used, with the Tournier iterative algorithm selected, to estimate a response function [37]. Finally, the dwi2fod routine was used to generate the fODF, using the constrained-spherical deconvolution (CSD) method [38].

### Automatic Segmentations of Regions of Interest

Estimation of regions of interests (ROIs) to seed and restrict the tractography algorithm was done by parcellation of the brain in cortical and sub-cortical areas. These areas were used to define the estimated regions of interest for seeding and restriction of the tractography algorithm. Spatially Localized Atlas Network Tiles (SLANT) uses deep learning to compute a subject-optimized whole brain segmentation of 133 anatomical regions (63 per hemisphere, 7 containing part of both hemispheres) [23]. Further refinement was achieved by the use of a thalamus segmentation algorithm available as a package in FreeSurfer, which uses a probabilistic atlas of the thalamus together with Bayesian inference to tailor the atlas to the individual subject [18, 39]. The thalamus segmentation algorithm was adapted to use, as an input, the segmentation results of SLANT in stead of the FreeSurfer results, so that it was not necessary to run the computationally heavy FreeSurfer segmentation algorithm. This led to a hybrid segmentation approach incorporating both DCNN and classical segmentation algorithms.

### Tractography Algorithm and Parameters

Tractography was performed using a probabilistic tracking algorithm available in MRTrix3. Specifically, the iFOD2 method was used, which is based on the fODF computed during preprocessing (c.f. the “Imaging Data and Preprocessing” section) and was chosen for its ability to resolve intra-voxel crossing fibers [40]. For each tractogram, streamlines originate from randomly placed voxels within the seed region and were generated, unidirectionally (i.e., growing only from one side), until a voxel of the target region was reached. When using multiple target regions, the algorithm was set to stop growing until any of these target regions had been reached. For further optimization, the protocol makes use of exclusion regions for four of the six tracts. Any streamlines entering an exclusion region were discarded.

Both the anatomical definitions of the ROIs that were used, as well as tractography parameters, were optimized for each tract separately. This was done on a (patient) case-by-case basis in close collaboration with clinical experts (among others authors GR and ML) and technicians (author SM). Important parameters that required tuning were the number of streamlines and the fODF amplitude cut-off value. The latter value determines the localized minimal signal-to-noise ratio that is sufficient for propagating streamlines.

### Anatomical Definitions of ROIs

The six tracts that we have chosen for this pipeline are all functionally important tracts as seen from a clinical-neurological perspective and have shown reasonable to good correspondence to results from intraoperative electrical stimulation mapping [32, 41]. ROIs were carefully selected on the basis of clinical expert knowledge and review of the literature. Before we conducted the actual experiments that are described in this paper, there was a developmental phase of approximately 2 years where we iteratively optimized the include and exclude regions of interest and several parameters. We did so by testing the pipeline in brain tumor patients and discussing results with a group of clinical experts, including neurosurgeons that perform awake tumor surgery in our hospital. We have ample experience in our center with the use of preoperative and (navigated) intraoperative tractography. In 2014, we published a protocol for four clinically relevant tracts (corticospinal tract, arcuate fasciculus, inferior fronto-occipital fasciculus, optic radiation) based on 100 brain tumor cases [31]. Over the years, this protocol was refined and expanded by us, based on state-of-the-art knowledge in the literature and clinical feedback from (awake) brain tumor cases, and was subsequently implemented in our automated pipeline [30, 32, 42]. Of note, results of our current experimental prototype pipeline are still regularly asked for by neurosurgeons from our hospital and considered a valuable adjunct to our existing clinical workflow for tractography [31].

#### Corticospinal Tract (CST)

The ipsilateral anterior part of the midbrain was used as a seed region to include the cerebral peduncle that contains the corticospinal tract. Since this anatomical region was not available in the SLANT atlas, it was created by (1) slicing the brain stem along its anterior–posterior axis, and selecting the anterior part, and (2) selecting an axial slice of 4 mm at the anatomical midpoint of the fourth ventricle. Target region is the precentral gyrus, including the more lateral parts (that are generally the most difficult part to reconstruct due to the high number of crossing fiber projections in that region). Additional refinements were made using the contralateral brainstem as exclude region, to reduce the number of false-positive streamlines that cross the midline of the brainstem, and the thalamus as exclude region (to discard false-positive streamlines within the thalamus). An anatomical feature of the corticospinal tract is that it contains fibers running from medial to lateral along the convexity of the precentral gyrus (primary motor cortex) all going downwards aligning in the anterior part of the brain stem.

#### Inferior Fronto-occipital Fasciculus (IFOF)

We chose as seed region the pars triangularis, pars opercularis and pars orbitalis of the inferior frontal gyrus, the anterior and posterior orbital gyrus, and the frontal pole (frontopolar cortex). The target regions selected in the current study were the inferior, medial, and superior occipital gyri. An anatomical feature of the inferior fronto-occipital fasciculus is that the fibers all traverse through the capsula externa. Therefore, this region was provided as an additional include region to the algorithm. The capsula externa was approximated by selecting a spherical region of 24 mm^3^ inside the white matter between the posterior insular cortex and the putamen.

#### Optic Radiation (OR)

As a seed region, the lateral geniculate nucleus (LGN) was used, with an additional constraint of the seeding direction laterally towards the temporal and frontal lobes in order to reduce the number of false-positive streamlines. In the MRTrix3 tractography algorithm, this was achieved by enforcing the initial seed direction within a 90°-cone centered in a specified direction. Furthermore, the LGN region was enlarged using a dilation operation with a Gaussian kernel (1.5 mm isotropic) to increase the likelihood of segmenting the LGN given anatomical variations in this area. The target region is the calcarine cortex (i.e., primary visual cortex). For refinement, we used the following additional exclude regions: the precuneus, basal forebrain, and the lingual gyrus. An anatomical feature of the optic radiation is Meyer’s loop, which has a high fiber curvature and often has an underestimated anterior extent due to interference from crossing fibers [43].

#### Arcuate Fasciculus (AF)

The terminology of the arcuate fasciculus (AF), especially in relation to the superior longitudinal system, is still under debate [44, 45]. An anatomical feature of the arcuate fasciculus is that arches around the insular cortex on a sagittal plane, distinct from the SLF III that has a more horizontal course and terminates in the supramarginal gyrus [45]. The seed regions consist of the pars triangularis and pars opercularis of the inferior frontal gyrus, and the ventral and inferior part of the precentral gyrus. The target regions are the middle and posterior part of the superior and medial temporal gyrus. For additional refinement, the putamen, anterior insula, and the thalamus were used as exclude regions.

#### Frontal Aslant Tract (FAT)

As seed regions, the supplementary motor area (SMA) and pre-supplementary motor area were used. As there are no anatomical landmarks that define the pre SMA, a vertical virtual plane passing through the genu of the corpus callosum was used to determine the anterior boundary of the pre SMA. The target regions are the pars opercularis and pars triangularis of the inferior frontal gyrus [46]. An anatomical feature of the frontal aslant tract is its oblique (aslant) course within the frontal lobe.

#### Superior Longitudinal Fasciculus (Third Component) (SLF)

The superior longitudinal fasciculus (SLF) is commonly divided into three distinct bundles based on their cortical seed and target regions [42]. In the current study, we investigated the ventral component, referred to as the SLF III. For this component, the seed regions are the pars triangularis and pars opercularis of the inferior frontal gyrus and the ventral precentral gyrus. The target region is the supramarginal gyrus. Additional exclude regions were placed at the angular gyrus, the thalamus and the superior temporal gyrus. The superior temporal gyrus was slightly enlarged using a dilation operation with a Gaussian kernel (3.0 mm isotropic) within the grey matter in order to reduce the number of false-positive streamlines. An anatomical feature of the SLF III is its horizontal course connecting frontal with parietal regions. It is located laterally to the superior limiting sulcus, whereas the AF arches around the insula and is located medially to it.

### Post-tractography Filtering

A downside of probabilistic tractography is that the resulting tractograms may contain spurious, or deviating, streamlines. These are noisy streamlines that are not well-aligned with their neighboring streamlines and likely have little anatomical significance. To produce white matter tracks that are more robust under the stochastic realizations of probabilistic tractography, we opted for a filtering based on the Fiber-To-Bundle Coherence (FBC) measures that are publicly available in the Diffusion Imaging in Python (DIPY) package [47, 48]. For an explanation of how to use the FBC measures, the reader is referred to https://tinyurl.com/FiberToBundle. The FBC measures compute a kernel density estimation for all the streamlines, in the space of both positions and orientations, and subsequently filter the fibers that have a particularly low density.

The main parameter of the FBC measures that requires careful tuning is the cut-off density (denoted by RFBC) of removing spurious fibers. We tuned this parameter for each tract individually, based on a visual inspection by medical experts, whether or not the filtering removed any anatomically plausible fibers, and always opting for a conservative setting. For visual inspection, both a cross-section of single subjects was investigated, as well as the results of a group-averaged tractogram map.

### Technical Specifications and Integration Into the Clinic

The pipeline was implemented in Python 3 and was segregated into a presentation layer, using Flask to serve a web-page for user management, and a data access layer, using the Flask REST-API together with a SQLite3 database. For optimal performance, the GPU-accelerated version of FSL Eddy, included in the dwipreproc script in MRTrix3, was used. ITK was used to generate a DICOM containing the tractography results in a color-coded voxelized image for export into a PACS system.

The pipeline was installed on a Debian 10.8 server equipped with an Intel Xeon Gold 6244 CPU with 32 threads, 251 GB of RAM, and two NVIDIA Quadro RTX 6000 videocards. The averaged processing time for all subjects was 2.2 ± 0.3 h. Note that since the preprocessed DWI images available in the HCP repository were used for all healthy volunteers, the FSL Eddy processing step was skipped.

### Experiments

#### Experiment 1: Tractogram Probability Maps

Tractogram probability maps make it possible to measure the variability of the tractography method and represent the probability of finding a streamline in a voxel. These group maps reflect variability from multiple sources, such as anatomical inter-subject variability (for instance reflected in significant differences in volumes of ROI) or non-deterministic noise factors from the probabilistic tractography algorithm. For each bundle of each subject, a tract density image was created using the tckmap function in MRTrix3, which counts the number of streamlines that traverses a voxel [49, 50]. Subsequently, this tract density image was transformed to the MNI-152 template by non-linear transformation using NiftyReg, using the DWI b = 0 image as a reference for computing the warp-field necessary for the transformation [51]. Each normalized tract density image was binarized by applying a threshold on the number of streamlines *K* such that voxels that satisfy *K* ≥ 1 become one and otherwise become zero. Finally, a tractogram probability map was computed by combining the normalized and binarized tract density images and averaging the result for each voxel.

To assess inter-subject anatomical variability, mean volumes and standard deviation of seed and target ROIs specified in the tractography protocol (see the “Anatomical Definitions of ROIs” section) were calculated, averaged for the left and right hemispheres individually.

#### Experiment 2: Hold-out Cross Validation

To quantify the robustness of the tractogram probability maps, a hold-out cross validation evaluation is applied. For this purpose, subjects are split into randomized testing and training groups, and the averaged similarity of both groups is repeatedly compared to each other. This is a model validation technique that is commonly used to evaluate how the results of an analysis will generalize to an independent data set. A low cross validation error indicates that a robust tractogram probability map can be created from a small subset of the data. While a priori variation is expected within the tractography results (as this is the rationale to perform subject-specific tractography in the first place), this procedure can nevertheless signal whether substantial deviations from the mean are present and thus identify potential false-positive outliers. The hold-out procedure in general involves the following steps: (1) creation of arbitrarily labelled training and testing groups of subjects, the relative group sizes of which are defined as the so-called hold-out ratio; (2) creation of tractogram probability maps for both groups according to the “Imaging Data and Preprocessing” section; (3) calculation of the overlap between the tractogram probability maps of both groups. Steps 1–3 are repeated *N* times, and the resulting overlap values are averaged. To compute the overlap, the Dice coefficient was used and calculated according to *O*_Dice_ = 2*N*(*A* ∩ *B*) / (*N*(*A*) + *N*(*B*)) where *A* and *B* are binarized tract density maps and *N* is the number of nonzero voxels in the map. In the current study the hold-out validation procedure was performed using a hold-out ratio of 10% and with 300 randomized subsets. Based on these parameters, random subsets of training (*N* = 13) and testing (*N* = 129) datasets were selected, after which binarized probability maps were generated using a 5% minimal confidence level.

#### Experiment 3: Repeated Processing of Identical DMRI Scans

To investigate the influence of the probabilistic tractography algorithm on single-subject tractograms, we randomly selected 10 subjects from the HCP dataset and applied the automatic tractography pipeline with identical settings 10 times for each subject. For each tract and each iteration individually, a binarized tract density map was created according to the procedure as described in Experiment 1: Tractogram Probability Maps. Finally, as a measure of reliability, the average Dice coefficient overlap was computed for each tract by taking all possible combinations (without repetition) between the iterations, i.e., $$\left(\begin{array}{c}10\\ 2\end{array}\right)$$ combinations per tract.

#### Experiment 4: Repeated Scanning of Subjects At Different Time Points

Repeated processing of identical scans is useful to establish an upper limit regarding the reproducibility of the pipeline. It does, however, not take into account the noise that results from changes in the hardware and physiology of the subject over time. To this end, we evaluate data of 40 subjects available in the HCP that were scanned twice at different time points. A pair-wise comparison of tractogram probability maps was performed by first normalizing the maps using a non-linear coregistration in subject-specific space, and subsequently calculating the overlap using the Dice coefficient. The distribution of overlap values for all 40 pair-wise processed subjects is then plotted. To investigate the effect of repeated scanning on segmentation of the ROIs, the Dice coefficient was calculated between pairs of seed or target regions.

## Results

### Tractography Algorithm and Parameters (End Results of Optimization Process)

The number of streamlines was set at 5000 streamlines for the FAT, 15,000 streamlines for the IFOF, 7000 streamlines for the SLF, and 7500 streamlines for the AF, CST, and OR. The fODF amplitude cutoff was set at 10% of the maximum amplitude for the OR, 7% for the IFOF and CST, 7.5% for the AF, 8% for the SLF, and 9% for the FAT. For all tracts, a step size of 0.2 mm and a radius of curvature of 1 mm were used, and the fODF was fitted with 8 spherical harmonic coefficients. The maximum number of times that the tracking algorithm was run to find an appropriate tracking direction was set at 10,000 attempts per seed voxel.

### Anatomical Definitions of ROIs

**Fig. 2:**
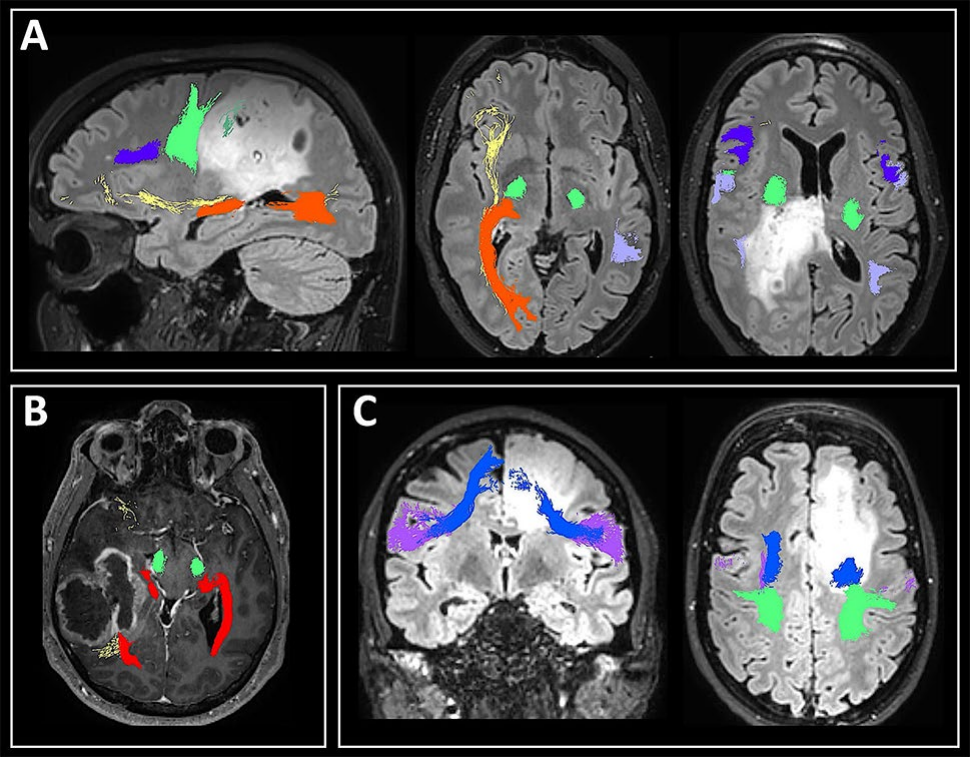
Results of the automated pipeline shown in three patients with gliomas, illustrating that in some patients tracts are displaced (e.g., the corticospinal tract (green) in patient **A** with a low-grade glioma, and the optic radiation (red) in patient **B** with a high-grade glioma), whereas in other patients tracts have infiltrated parts of the tumor (e.g., the frontal aslant tract (blue) in patient **C** with a low-grade glioma). For these patients, MR-based tracts were in accordance with clinical findings and results of intraoperative electrical stimulation mapping

Approximately 50 patients with low-grade and high-grade gliomas were analyzed, whereby ROIs were iteratively optimized based on feedback of neurosurgeons who specialize in (awake) brain tumor surgery and functional neuroanatomy. Resulting seed, target and exclude regions of interest were used for the current study in healthy subjects. Figure 2 shows some illustrative patient examples.

#### Experiment 1: Tractogram Probability Maps

**Fig. 3:**
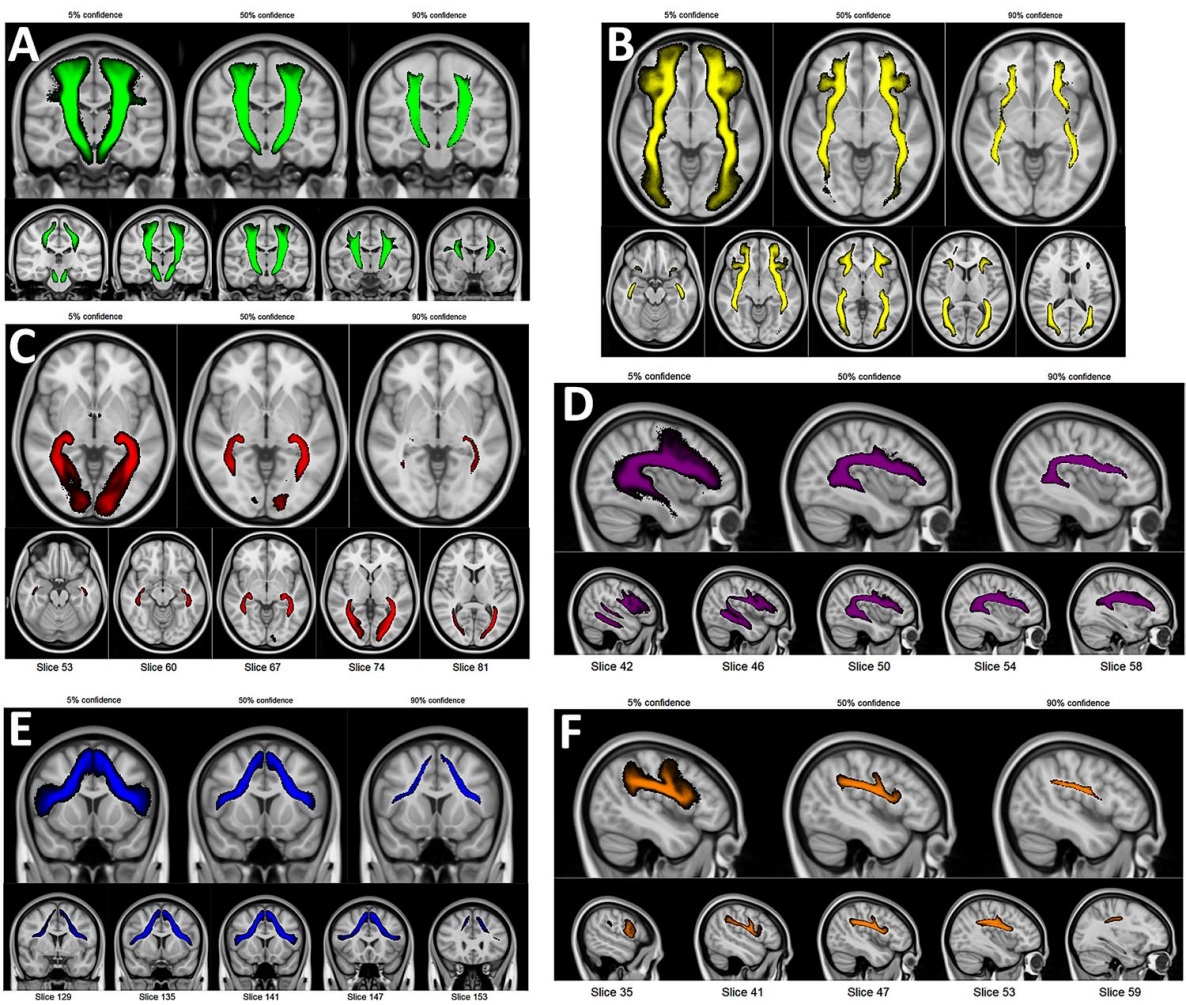
The tractogram probability map shown for six white matter tracts as visualized on the MNI-152 anatomical template; **A** corticospinal tract, **B** inferior frontal occipital fasciculus, **C** optic radiation, **D** arcuate fasciculus, **E** frontal aslant tract, **F** superior longitudinal fasciculus, third branch. For each panel, the top row depicts maps with a varying minimal confidence level, showing all the voxels where at least in 5% (respectively 50% and 95%) of cases a streamline appeared in each voxel. For each panel, the bottom row depicts brain slices showing the tractogram probability map at a 50% confidence level. Each map is color-coded and scaled by brightness from minimal to maximal confidence. See also Fig. 7 in Appendix for more details on the optic radiation

Tractogram probability maps were generated for the six tracks of interest and are illustrated in Fig. 3 (see for further details of the optic radiation also Fig. 7 in Appendix).The tractogram probability maps are shown for varying minimal confidence levels (5%, 50%, 90%), indicating, for each voxel, the percentage of test subjects whose tract density image contained at least one streamline. The maps have been made publicly available in NIftI-1 format at the following URL: https://tinyurl.com/ TractographyPipelineMaps.

The average standard deviation for the seed and target ROIs amounts to 26% and 23%, respectively. This indicates that there is considerable anatomical variation in the size of the ROIs.

#### Experiment 2: Hold-out Cross Validation

**Fig. 4:**
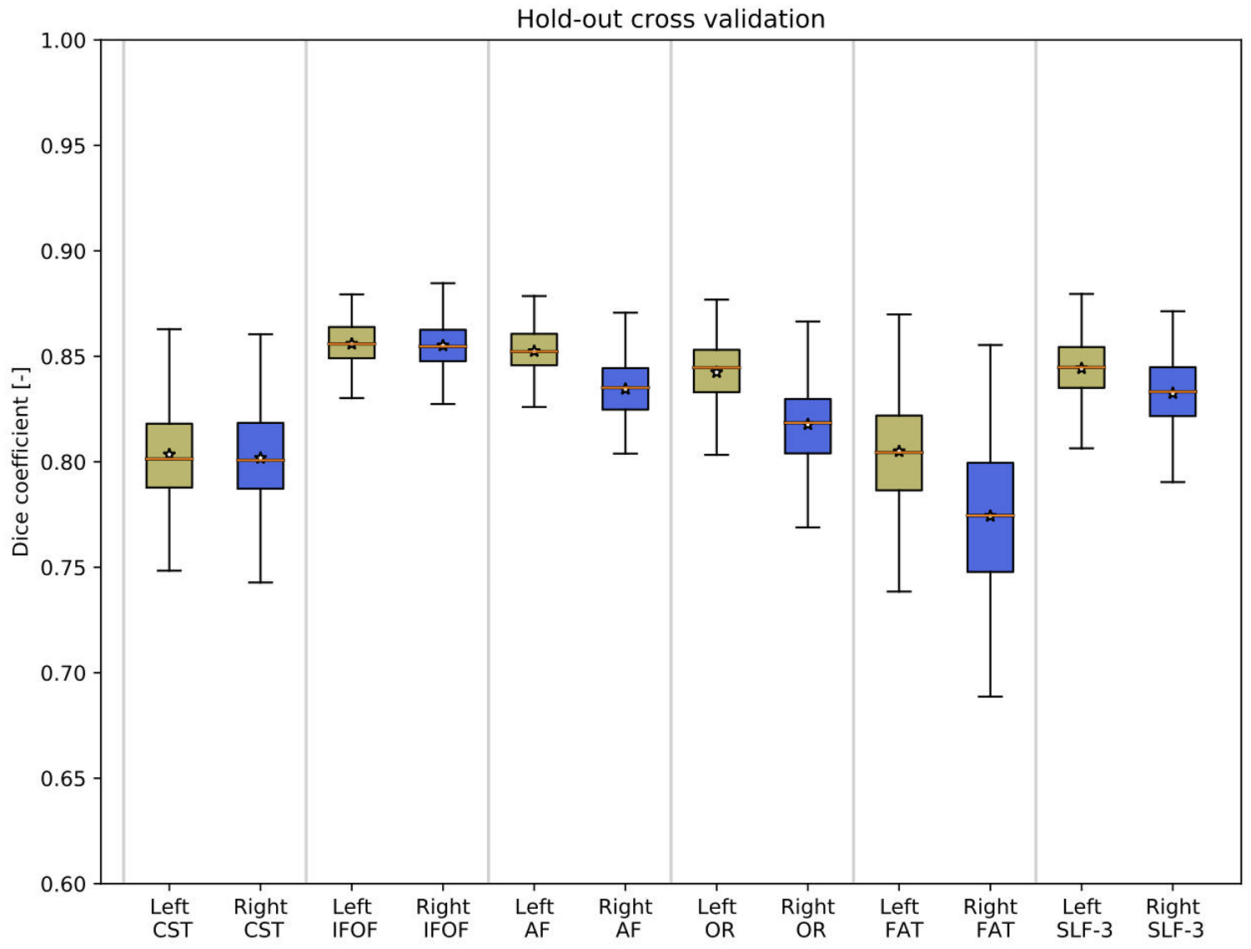
Estimation of the robustness of the pipeline via tractogram probability maps using holdout cross validation, shown as box-and-whisker plots. Of the available datasets (*N* = 136), randomized testing and training sets were created (300 iterations) using a 10% hold-out rate. Most tractograms indicate a moderately good average overlap of testing and training maps, at a Dice coefficient over 0.7

The results of the hold-out validation procedure are shown in Fig. 4, indicating for each tract the average Dice coefficient between randomized testing and training maps in the form of a box and whisker plot. The lower extremes of the box and whisker plots show for all tracks a Dice coefficient at or above 0.7, which is traditionally considered a good overlap value, indicating that there are no substantial deviations in overlap from the mean [52]. The average Dice coefficient is near 0.8 for all tracts considered, indicating a moderately high degree of overlap between randomized testing and training maps.

#### Experiment 3: Repeated Processing of Identical DMRI Scans

**Fig. 5:**
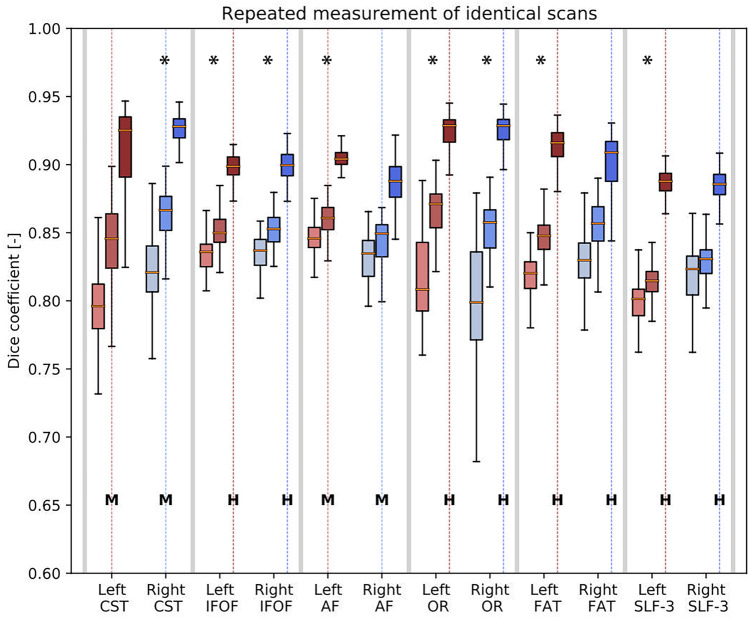
Estimation of the reliability of repeated processing of dMRI scans. Shown for different stages of filtering from left to right: none, medium (RFBC = 10 − 3) and high (RFBC = 10 − 1) using box-and-whisker plots. In most cases, the use of medium filtering led to an increase in the Dice coefficient, and thus the inferred stability of the tracts. The high filtering further increased the Dice coefficient further for all tracts. Statistical differences are indicated with an asterisk

In Fig. 5, the results of repeated processing of identical dMRI scans are shown. For each tract, the reliability after the application of FBC filtering (see “Post-tractography Filtering” section) is indicated using either none, medium (RFBC = 10^−3^), or high (RFBC = 10^−1^) filtering settings. The filtering setting used in the tractography protocol is indicated for each tract by M (medium) or H (high). It can be observed that, for each tract, the Dice coefficient increased as the post-tractography filter was applied with a stronger filter setting. Using the strongest filter setting, the Dice coefficient was near or above 0.9 in all cases.

#### Experiment 4: Repeated Scanning of Subjects At Different Time Points

**Fig. 6:**
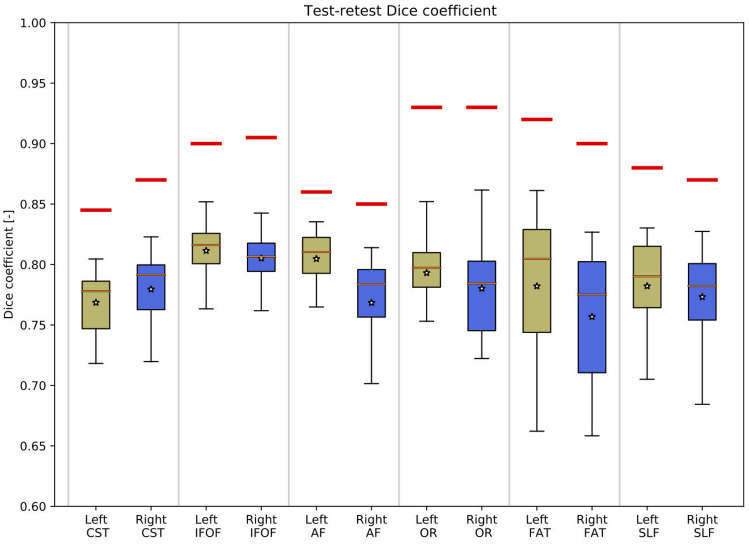
Demonstration of the reliability of the tractogram probability maps in a pair-wise evaluation of the test–retest data of 40 healthy subjects, shown as box-and-whisker plots. Each tract is color-coded as khaki (left hemisphere) and blue (right hemisphere). For each tract, the reliability as determined from the processing of identical dMRI scans (see Fig. 4) is plotted as a red line

**Fig. 7:**
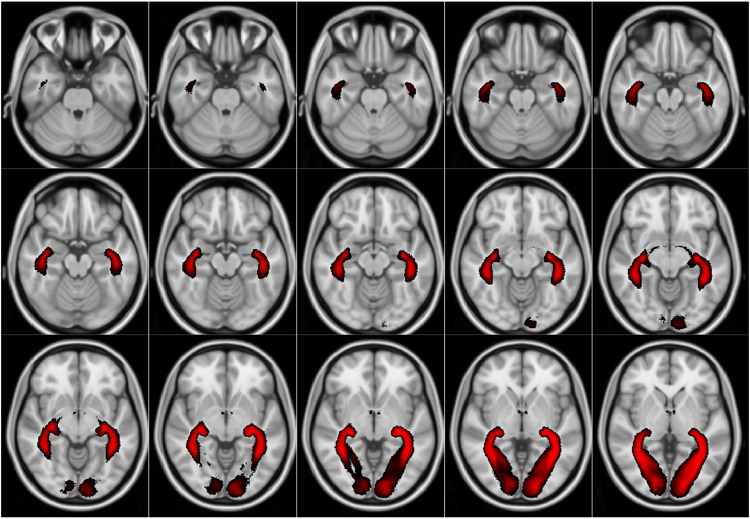
The tractogram probability map shown for the optic radiation using a 5% minimal confidence level. Each slice is now sampled 2 mm apart to better show Meyer’s loop in the anterior part of the temporal lobe. Each map is color-coded and scaled by brightness from minimal to maximal confidence

The results of the test–retest variability assessment are shown in Fig. 6. The Dice coefficients indicate a good average coherence (above 0.75), indicating that the tractography results have a high reliability when considering variations in input data between two time points that cannot be controlled for. For each tract, baseline reliability as estimated from the processing of identical dMRI scans (see Experiment 3: Repeated Processing of Identical DMRI Scans) is plotted as a red line, which corresponds to the average Dice coefficient for the FBC filtering strength selected as part of the tractography protocol. The variability from the repeated scanning experiment is understandably lower, but close to, the reported upper limit.

Test–retest results for the automated segmentation of ROIs is listed in Table 1. For each subject, the Dice coefficients of the left and right hemispheres were averaged. All coefficients were above 0.8, indicating a moderately high overlap.

**Table 1:** Dice coefficient calculated between pairs of seed or target regions of interest from the test–retest dataset

ROI	CST	IFOF	OR	AF	FAT	SLF III
Seed left	0.93 ± 0.02	0.84 ± 0.02	0.77 ± 0.02	0.84 ± 0.02	0.86 ± 0.02	0.84 ± 0.02
Target left	0.83 ± 0.02	0.86 ± 0.02	0.87 ± 0.03	0.89 ± 0.02	0.84 ± 0.03	0.85 ± 0.03
Seed right	0.93 ± 0.02	0.84 ± 0.03	0.76 ± 0.08	0.84 ± 0.02	0.85 ± 0.03	0.84 ± 0.03
Target right	0.83 ± 0.03	0.87 ± 0.02	0.87 ± 0.03	0.89 ± 0.02	0.85 ± 0.03	0.84 ± 0.03

## Discussion

Clinicians today lack a standardized method for acquiring and reconstructing MRI-based white matter tracts due to various methodological, physiological and pathological sources of variability, leading to a large inter-user variability in the interpretation of results [1]. This raises a chicken-and-egg problem because as long as there is no consensus on methodology and definition of tracts among different users and centers, results will a priori differ, complicating clinical validation. Even among knowledgeable and experienced users, there is concern about intra- and interuser reproducibility with manual placement of ROIs [53]. We developed a modular data-processing pipeline that allows for the fully automatic reconstruction of white matter tracts to address this problem. ROIs were carefully selected and iteratively optimized on the basis of clinical expert knowledge and review of the literature. We subsequently investigated robustness and reliability (i.e., test–retest reproducibility) of the pipeline in healthy subjects for six clinically relevant tracts. We see our preliminary work as a first and necessary step toward validation and subsequent clinical use of tractography in brain tumor patients.

Robustness of tractograms was evaluated using a holdout cross validation approach, and indicated that there were no substantial deviations from the mean in terms of overlap. Group maps of each tract yielded a consistent anatomical course, suggesting a low rate of false-positives and indicating a high robustness of tractography results. Although there is obviously some inter-subject variability due to differences in anatomy, as demonstrated also by the variability in sizes of the individual ROIs, there were no major outliers [54]. Reliability was evaluated in two ways: by repeated processing of identical MRI data sets along the pipeline (which estimated test–retest properties of the probabilistic algorithm), and by test–retest evaluation of subjects at different points in time (this further added variability due to differences in noise due to hardware and subject-specific physiology). Repeated processing of identical MRI scans showed a high overlap (average Dice coefficient of 0.9 for all tracts) without any post-tractography filtering. When post-tractography filtering is applied, reliability increases significantly when the strength of the filter is increased. The amount of filtering chosen, which was either of the medium or high variant, was based on visual inspection regarding the presence of streamlines that were deemed false-positives. In these cases, streamlines would for example enter anatomically implausible regions. In the current study, we have opted for medium and high filtering variants to find a balance between removing spurious streamlines and retaining true positive streamlines.

Test–retest characteristics of methods to derive white matter tracts have received significantly less attention than for other neuroimaging techniques [55]. We know of no studies that analyzed robustness and test–retest reproducibility of fully automatized subject-specific tractography in healthy subjects. Kristo et al. performed a test–retest study in eleven healthy subjects with manually-selected ROIs. These authors found a mean overlap between sessions (as calculated with a Dice score) of 0.63 for the corticospinal tract and 0.58 for the arcuate fasciculus. These numbers are somewhat lower than ours (> 0.75 for all tracts), perhaps reflecting additional uncertainty due to manual segmentation. Boukadi et al. used a “nearly automatic way” to extract white matter bundles of the language network in eighteen healthy subjects, and describe a variable, but overall good overlap between two time points, with (weighted) Dice coefficient over 0.70 for all studied language tracts [55]. Tracts were reconstructed using White Matter Query Language, a method developed by Wassermann et al. that uses a dictionary of definitions including gray and white matter regions and rules for spatial relations [56].

A major factor in the reliability of tractography results is the segmentation quality of the anatomical regions used [53]. A novelty of our approach is that we incorporated a deep convolutional neural network in our automated pipeline (DCNN). This method makes it possible to delineate (sub)cortical structures and ROIs at the level of the individual subject, as opposed to the traditional atlas-transformation-based approaches that are based on group averages. This method potentially better addresses the significant variability in sulcal and gyral anatomy that is present in normal subjects [57]. Size of the ROIs in our study indeed varied considerably between subjects whereas intrasubject variability was low, stressing the importance of a subject-specific approach and arguing against the use of standardized atlases. Notably, in patients with brain tumors, this variability will increase dramatically due to mass effect or infiltative growth of the tumor, underlining the need for an individualized approach for ROI identification in patients.

In the current study, the SLANT algorithm, which is a DCNN-based brain segmentation algorithm, was used to segment the ROIs used in the tractography protocol. SLANT was one of the first algorithms to provide a whole-brain segmentation with over 100 labels, which was a technical challenge due to restrictions in graphics processing unit (GPU) memory, and provided superior results to other U-net-based DCNN algorithms at the time [23]. Assemblynet provides an extension to the SLANT algorithm by using a larger number of more compact 3D U-Nets and yielded improved results in segmentation consistency and accuracy; however, its implementation is not publicly available [26]. PSACNN and FastSurfer have possibly improved segmentation performance but offer substantially fewer segmentation labels [25, 27]. ACEnet is a recently released DCNN segmentation algorithm that offers as many labels as SLANT and indicates a higher segmentation performance than both SLANT and AssemblyNet [28]. Additionally, ACEnet offers an open-source implementation, which makes it a very promising replacement candidate for the SLANT algorithm used in the current work.

### Limitations and Future Research

The post-tractography filtering with the FBC filtering algorithm required tuning of the cut-off density, or RFBC parameter, for removing spurious fibers. The optimization of this filtering parameter remains an unresolved issue due to the absence of a ground truth in tractography results. An indirect approach for optimizing the filtering parameter was explored in the specific case of temporal lobe epilepsy surgery in a previous study from our group, where pre- and post-operative comparisons of tractography results could be related to the extent of resection [47]. An approach that has emerged in recent years, and potentially could be useful for tuning tractography results in patients, is comparing the results of tractography to intraoperative electrical stimulation mapping [58–60]. This method is considered the clinical gold standard for the identification of functional white matter tracts. Although it certainly also has its flaws, in general, good correspondence has been noted for selected motor and language tracts between both methods [61].

Creating reliable tractography results becomes significantly more difficult when considering patients with neurological diseases, as opposed to healthy volunteers. Patients with a malignant brain tumor, for example, have altered brain physiology and anatomy due to mass effects and infiltrative growth within healthy brain structures. As a result, white matter tracts can be considerably deformed due to mass effects, which can make the task harder for a tractography algorithm due to for example increased curvature or fanning of axons, or due to edema. Perhaps even more significant is the deformation of cortical and subcortical structures. Our preliminary experience of the current pipeline in approximately 50 glioma patients indicated that the protocol based on DCNN performed very well in the presence of tumorous masses (qualitative judgement made by clinicians, data not published in this paper). We therefore expect a significant gain when compared to more standard anatomical atlas-based approaches that assume an underlying healthy brain, but this needs to be formally tested and is still a potential limitation of our current pipeline. Future work has to focus on the evaluation of the whole automatic tractography pipeline in a clinical setting, evaluating its performance on patients with brain tumors. We also hope that different neurosurgical centers are able to use the pipeline, so tractography results can be validated and further optimized in a multi-center setting, speeding up the process of broad clinical use and acceptance.

## Conclusion

The current study presents an automatic tractography pipeline that is able to generate robust and reliable and subject-specific white-matter reconstructions in healthy subjects. This was accomplished using the DCNN approach for detailed subject-specific cortical and subcortical segmentations, and post-tractography filtering to reduce the number of false-positive streamlines. As a next step, we are planning to validate our reliable and timely subject-specific pipeline in a systematic manner in a cohort of brain tumor patients, whereby we also aim to introduce the pipeline in other centers to initiate further optimization in a multi-center setting.
